# The Effect of TLR9 Agonist CpG Oligodeoxynucleotides on the Intestinal Immune Response of Cobia (*Rachycentron canadum*)

**DOI:** 10.1155/2014/273284

**Published:** 2014-06-02

**Authors:** Omkar Byadgi, Dinda Puteri, Jai-Wei Lee, Tsung-Chou Chang, Yan-Horn Lee, Chun-Yen Chu, Ta-Chih Cheng

**Affiliations:** ^1^Department of Tropical Agriculture and International Cooperation, National Pingtung University of Science and Technology, Pingtung 91201, Taiwan; ^2^Department of Fisheries and Marine Science, University of Brawijaya, Malang 65145, Indonesia; ^3^Anatomy & Pathology Laboratory, Department of Veterinary Medicine, National Pingtung University of Science and Technology, Pingtung 91201, Taiwan; ^4^Tungkang Biotechnology Research Center, Fisheries Research Institute, Council of Agriculture, Pingtung 91201, Taiwan; ^5^Graduate Institute of Animal Vaccine Technology, National Pingtung University of Science and Technology, Pingtung 91201, Taiwan

## Abstract

Cytosine-guanine oligodeoxynucleotide (CpG ODN) motifs of bacterial DNA are recognized through toll-like receptor 9 (TLR9) and are potent activators of innate immunity. However, the interaction between TLR9 and CpG ODN in aquatic species has not been well characterized. Hence, cobia TLR9 isoform B (RCTLR9B) was cloned and its expression and induction in intestine were investigated. RCTLR9B cDNA consists of 3113bp encoding 1009 amino acids containing three regions, leucine rich repeats, transmembrane domain, and toll/interleukin-1 receptor (TIR) domain. Intraperitoneal injection of CpG ODN 2395 upregulated RCTLR9 A and B and MyD88 and also induced the expressions of Mx, chemokine CC, and interleukin IL-1**β**. Cobia intraperitoneally injected with CpG ODN 1668 and 2395 had increased survival rates after challenge with *Photobacterium damselae* subsp. *piscicida*. In addition, formulation of CpG ODN with formalin-killed bacteria (FKB) and aluminum hydroxide gel significantly increased expressions of RCTLR9 A (50 folds) and B (30 folds) isoforms at 10 dpi (CpG ODN 1668) and MyD88 (21 folds) at 6 dpv (CpG ODN 2395). Subsequently, IL-1**β** increased at 6 dpv in 1668 group. No histopathological damage and inflammatory responses were observed in the injected cobia. Altogether, these results facilitate CpG ODNs as an adjuvant to increase bacterial disease resistance and efficacy of vaccines in cobia.

## 1. Introduction

Teleost innate immunity plays an important role in the initial protection against invading pathogens [1]. The gastrointestinal (GI) tract is generally recognized as an organ crucial not only to the digestion/absorption of nutrients, but also to the immunity [2]. The GI tract serves as an important barrier and protects the fish from feed-borne pathogens [3–5]. The posterior segment of fish intestine is immunologically active and is armored with various immune cell types, including B cells, macrophages, granulocytes, and T cells, that play vital roles in local immune responses during the course of immunization and inflammation [6–8]. In addition, the fish intestinal epithelial cells are constantly exposed to pathogens and are involved in the innate immunity of GI tract. These cells recognize pathogen-associated molecular patterns (PAMPs) through the toll-like receptors (TLRs) and induce immune responses in the intestinal lumen [9].

TLRs are transmembrane proteins recognizing conserved pathogenic structures and activating immune effector molecules [10] to form a linkage between innate and adaptive immunity [11]. The main immune functions of TLRs are (1) inducing the expressions of pro/anti-inflammatory, cyto- and chemokine that link to the adaptive immune system; (2) initiating antimicrobial effects or pathways; and (3) maintaining commensal and mucosal homeostasis [12]. The role that toll-like receptor 9 (TLR9) plays in the innate immune responses to bacterial and synthetic DNA containing unmethylated CpG motifs has been well characterized [13]. The human TLR9 gene can splice into different isoforms during transcription generating 5 TLR9 isoforms (TLR9A, B, C, D, and E). These TLR9 isoforms are differentially expressed in various immune organs and cells, such as spleen, peripheral blood mononuclear cells (PBMC), and lymph nodes, which may attribute to differential profiles of proinflammatory cytokines and cytotoxic T-lymphocyte differentiation [14–16]. In teleost, only sea bream and croaker TLR9 B isoforms have been identified, but the functions of TLR9 B isoforms remain unclear [10, 17].

Cytosine phosphate-guanine (CpG) oligodeoxynucleotides (ODNs) are DNA fragments with a high frequency of CpG motifs simulating the immunostimulatory activity of bacterial DNA [18, 19]. CpG ODNs, unlike most conventional adjuvants, are able to stimulate both humoral and cell-mediated immune responses in immunized animals [20]. The interaction of TLR9 with CpG motifs initiates a cascade of events resulting in the secretion of T helper (Th) 1-type cytokines and chemokines. Productions of the chemokine and interferon-gamma-inducible protein-10 (IP-10) are early indicators of Th1 type immune responses [21, 22]. In mammals, intraperitoneal administration of CpG ODNs is effective in stimulating the expressions of intestinal TLR9 and chemokines due to the lower interleukin (IL)-10 level in human neonates and pig intestine [23, 24]. In teleost, administration of CpG ODNs has been found to increase TLR9 expression in Atlantic salmon [25], sea bream [26], and turbot [27]. However, to our best knowledge, the immune responses involved in the gut immunity in response to intraperitoneal stimulation of CpG ODNs in cobia have not been investigated.

The cobia industry has been suffering from various infectious diseases associated with* Photobacterium damselae* (*P. damselae*) subsp.* piscicida* [28–31] that has a high binding and invading capacity to the epithelial cells of fish intestine [32, 33]. Hence, the immunostimulatory effects of CpG ODNs might be able to enhance the intestinal immunity of cobia and increase the resistance to infections caused by* P. damselae* subsp.* piscicida.* The objectives of this study are to (1) clone RCTLR9B; (2) analyze the expressions of RCTLR9A RCTLR9B, MyD88, Mx, IgM, chemokine CC, and IL-1*β* in response to the stimulation of CpG ODNs; (3) evaluate the protection efficiency of intraperitoneal administration of CpG ODNs against* P. damselae* subsp.* piscicida* infections; and (4) assess the CpG adjuvanticity by intraperitoneal injection of CpG ODNs formulated with formalin-killed bacteria (FKB) and aluminum hydroxide gel (alum).

## 2. Materials and Methods

### 2.1. Cloning of Cobia TLR9 Isoform B

#### 2.1.1. Partial Cloning of Cobia TLR9 Isoform B

Partial sequence of cobia TLR9B was obtained using the primers (TLR9F1 and TLR9R1) which were designed based on TLR9 sequences of other fish species (*Acanthopagrus berda* number EU256332,* Dentex tumifrons* number EU256335,* Larimichthys crocea* A number EU655704,* Larimichthys crocea* B number EU655705,* Sparus aurata* A number AY751797, and* Sparus aurata* B number AY751796) using Vector-NTI (Invitrogen, Carlsbad, CA, USA) [34]. PCR was carried out using cobia cDNA from the liver as a template under the following condition: one cycle at 95°C for 2 min followed by 35 cycles of 95°C for 30 s, 54°C for 30 s, and 72°C for 1 min; and a final extension at 72°C for 5 min. PCR products were sequenced and used for designing real-time PCR primers.

#### 2.1.2. Rapid Amplification of cDNA Ends (RACE)

To obtain the full-length cDNA sequence of RCTLR9 isoform B (RCTLR9B), 5′ and 3′ RACE were implemented using the SMARTer RACE cDNA amplification kit (Clontech, USA). The RCTLR9 gene primers, TLR9B-RacF1 and TLR9B-RacR1, were designed from the partial RCTLR9B cDNA sequence (JX073035.1). Touchdown-PCR was carried out for 3′ and 5′ RACE using TLR9B-Rac F1/UPM and TLR9B-RacR1/UPM primer sets (Table 1), under the following conditions: one cycle of initial denaturation at 94°C for 2 min followed by 5 cycles of 94°C/30 s, and 72°C/3 min; next 5 cycles 94°C/30 s, 70°C/30 s, and 72°C/3 min; next 25 cycles 94°C/30 s, 68°C/30 s, and 72°C/3 min; and one cycle at 72°C/5 min. Nested PCR for 5′ and 3′ RACE was performed using TLR9B-NGSP1/NUP for 5′ and TLR9B-NGSP2/NUP for 3′ nested primer sets (Table 1). For nested PCR, 1 *μ*L of primary RACE-PCR product was used as a template with the following conditions: initial one cycle of 94°C/2 min and then 30 cycles of 94°C/30 s, 68°C/30 s, and 72°C/3 min followed by one cycle of 72°C/5 min. The PCR products were sequenced and analyzed using Vector-NTI program and BLASTx was performed using NCBI website (http://www.ncbi.nlm.nih.gov/blast/) on the GenBank database.

#### 2.1.3. Analysis of TLR9 cDNA Sequences

The full-length cDNAs of RCTLR9B were assembled using vector NTI program. The deduced amino acid sequence, molecular weight (kDa), pI, and protein analysis were conducted by the ExPASy proteomic tool (http://www.expasy.org/tools/). The protein domains were predicted using SMART software (http://smart.embl-heidelberg.de/). Homologous sequences were searched using BLAST program available at the NCBI website with default settings on the GenBank database. The multiple sequence alignment was created using the CLUSTALW (http://www.ebi.ac.uk/clustalw2/).

### 2.2. CpG ODNs

CpG ODNs were purchased from Bioneer (Korea). The ODNs were phosphorothioate modified throughout the sequence. Sequences of CpG ODNs are as follows:

 B-Class CpG 1668 T∗**C∗G**∗T∗**C∗G**∗T∗T∗T∗T∗G∗T∗**C∗G**∗T∗T∗T∗T∗G∗C∗T∗G,
 C-Class CpG 2395 T∗**C∗G**∗T∗**C∗G**∗T∗T∗T∗T∗**C** **∗G**∗G∗**C∗G**∗**C∗G**∗**C∗G**∗C∗**C∗G** and 2137 (control CpG with phosphorothioate modified inverted),
 T∗**G∗C**∗T∗**G∗C**∗T∗T∗T∗T∗G∗T∗**G∗C**∗T∗T∗T∗T∗G∗T∗**G∗C**∗T∗T (phosphorothioate modifications are marked with ∗ and CG and GCs are in bold).

The CpG ODNs were suspended in phosphate buffer saline (PBS, pH 7.2) and stored at −20°C until used.

### 2.3. Experimental Design and Fish Sampling

Cobia fish (approximately 20 g body weight) were procured from a local farm and they were maintained in recirculatory aerated tanks for acclimatization up to one week. During acclimatization, they were fed with a commercial diet and proper water quality (water level 200 L, temperature 28°C, and salinity 30 ppt) was maintained. Fish were randomly divided into four groups and each tank was stocked with ten fish (25 g). Fish were intraperitoneally (i.p.) injected with 0.5 mL PBS for the control group and 0.5 mL PBS containing 10 *μ*g unmethylated CpG ODN 1668, 2395, and 2137, respectively. At each time interval (1, 3, 6, and 10 days after stimulation), two fish were sampled and the posterior part of the intestine was hygienically dissected and immersed in PBS (pH 7.2). After the mucus and digested feed inside the intestine were completely removed, the samples were immediately immersed in TRIzol reagent (Invitrogen, USA). Total RNA isolation of the intestine was carried out according to manufacturer's instruction with minor modifications. The RNA pellet was dissolved in Rnase free water (Qiagen) and preserved in −80°C until used.

### 2.4. Protection Efficiency of Intraperitoneal Administration of CpG ODNs against Bacterial Challenge in Cobia

Cobia fish (20–25 g) were divided into five groups (*n* = 10 each) and acclimatized for three days. During acclimatization, fish were fed with a commercial feed daily. After three days, cobia were injected intraperitoneally (i.p) with 0.5 mL of PBS and 0.5 mL PBS containing 10 *μ*g CpG ODNs 1668, 2395, and 2137. After 48 h, cobia were challenged intraperitoneally (i.p) with live* P. damselae* subsp.* piscicida* bacteria (LD_50_—3.25 × 10^6^ CFU/mL). Thereafter, the water quality was properly maintained by vigorous aeration and by monitoring constant water temperature and salinity (28°C and 30 ppt). The behavior and mortality after challenge were recorded daily in individual group.

### 2.5. The Adjuvanticity of CpG ODNs

#### 2.5.1. Preparation of Bacterial Antigens

Bacteria were prepared from the frozen stock using the following conditions:* P. damselae* subsp.* piscicida* (BCRC 9714) was cultured in 5 mL brain heart infusion (BHI) broth containing 2% NaCl at 28°C/overnight and then 1 mL broth of the stock was transferred to 100 mL broth and grown until the O.D reaches 1.0 (600 nm). The bacteria were harvested by centrifugation (6000 ×g) at 4°C for 5 min. The pellet was washed twice in PBS (pH 7.2) and the bacterial suspension was inactivated by adding formalin to a final concentration of 3% and incubated overnight at 4°C. The inactivated bacterial solution was centrifuged at 6000 ×g for 10 min and thoroughly washed 3 times to remove formalin residues. The inactivation of FKB was confirmed by plating 100 *μ*L of solution on BHI + 2% NaCl plates and incubated at 28°C overnight.

#### 2.5.2. Formulation of CpG ODNs with FKB and Alum

Each dose (100 *μ*L) of vaccine contained 10 *μ*g of CpG ODNs (1668, 2395, or the control 2137) in 10 *μ*L PBS formulated with 45 *μ*L of 2% alum (Alhydrogel, Invivogen) and 45 *μ*L of FKB. Formulations were mixed at room temperature on a shaker at approximately 30 rpm for 30 min and stored at 4°C until used.

#### 2.5.3. Immunization and Sampling

All cobia were procured from a local fish farm in Pingtung, Taiwan, and were acclimatized as previously described. After acclimatization, twenty fish per tank were stocked into four different groups. The first group was immunized intraperitoneally (i.p.) with 100 *μ*L PBS. The second group was immunized with 100 *μ*L/fish vaccine containing 10 *μ*g/10 *μ*L of 1668 CpG ODNs plus 45 *μ*L FKB. The third (FKB + alum + CpG ODN 2395) and fourth groups (FKB + alum + 2137) were immunized with 45 *μ*L of 2% alum designated as FKB + alum + CpG ODN 1668. All formulations were injected in a total volume of 100 *μ*L/fish. After injection, the behavior of fish was observed daily and the optimal conditions were maintained. For gene expression studies, the posterior intestine was sampled for total RNA isolation at 1, 3, 6, and 10 days after injection (dpi) as described in Section 2.3.

#### 2.5.4. Histopathology

Two fish from each group were sacrificed at 3, 6, and 10 dpi, respectively, for histopathological examination. Tissue samples from the posterior part of the intestine were fixed in 10% neutral buffered formalin for standard procedures of light microscopy. Samples were processed, paraffin-embedded (Tissue-Tek TEC), and cut into 4-5 *μ*m sections using standard microtome (Leica RM2235) before being stained with hematoxylin and eosin (Tissue-Tek DRS). Stained slides were examined (Olympus) for signs of inflammation, dilation of lamina propria, and necrosis of epithelial cells using a double blind design. Slides were recoded to avoid observation bias before they were sent to the pathologist for further examination.

The sign of inflammation was scored from 0 to 3 (0 = normal, 1 = mild, 2 = moderate, and 3 = severe) [35]. Dilation of lamina propria, necrosis of epithelial cells, atrophy, deposits, serosa necrosis, submucosal necrosis, hypertrophy, and hyperplasia were also evaluated based on the histopathological changes as (−) no histopathology, (+) mild histopathology, (++) moderate histopathology, and (+++) severe histopathology.

### 2.6. cDNA Synthesis and Quantitative Gene Expression

The quality of total RNA was analyzed by a spectrophotometer using 260/280 nm UV. First strand cDNA was synthesized by reverse transcriptase on 2 *μ*g of total RNA using MULV reverse transcriptase enzyme (Lucigen, USA) with an oligo-dT primer. All the cDNA samples were stored at −20°C until used. The genomic contamination and quality of cDNA synthesis were determined by using PCR-amplification by designing primers on intron-exon flanking region and 3′UTR region of cobia *β*-actin3 (HM754627). The expression levels of TLR9 A and B (KC180322 and JX073035), MyD88 (KF018033), IL-1*β* (AY641829), Mx (AY834185), IgM (JX025102), and CC-chemokine (JF975593) were analyzed by gene specific primers (Table 1). Real-time PCR was carried out in an ABI 7500 real-time detection system (Applied Biosystems, USA). The amplification was performed in a total volume of 10 *μ*L, containing 5 *μ*L of SYBR green I real-time PCR master Mix (Kapa Biosystems), 1 *μ*L of cDNA, 0.2 *μ*L of each primer, and 3.6 *μ*L of DEPC water. The real-time PCR program was 95°C for 1 min followed by 40 cycles of 95°C for 15 s, 60°C for 60 s. Dissociation and melting curve of amplification products was performed at the end of each PCR to confirm that only one PCR product was amplified and detected. After amplification, data acquisition and analysis were conducted using the sequence detection software (SDS version 2.1. Applied Biosystems). The 2^−ΔΔCT^ method was chosen as the calculation method [36]. The difference between the cycle threshold (Ct) value of the target gene and the reference gene (*β*-actin) called ΔCT was calculated. ΔΔCT = (ΔCT of target gene or PBS-injected group for the target gene at each time point) − (ΔCT of the initial control).

### 2.7. Statistical Analysis

Data were analyzed using the SPSS16 (SPSS Inc., Chicago IL). Data distribution was determined using descriptive statistics. Differences in the means among the treatments were analyzed using ANOVA and compared using post hoc multiple comparison using Duncan's multiple range test. A *P* value of <0.05 was considered significant.

## 3. Results

### 3.1. Cloning of Cobia TLR9 Isoform B

**Figure 1:**
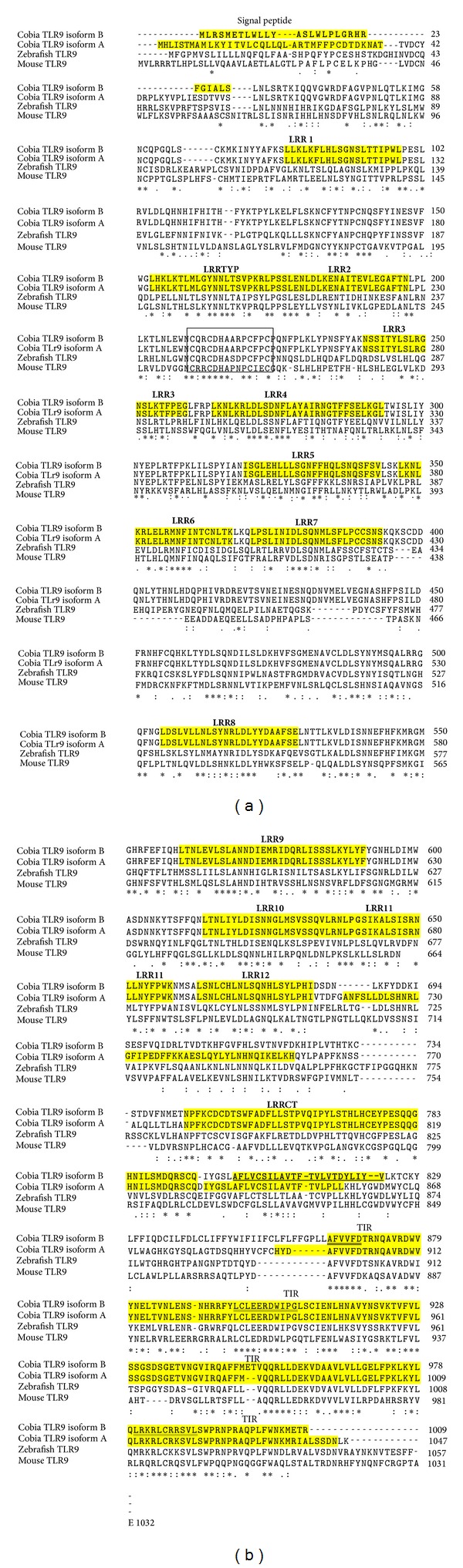
Amino acid sequence alignment of cobia (*Rachycentron canadum*) TLR9 isoform B deduced protein to cobia TLR9 (*Rachycentron canadum*, AGD79973.2), zebrafish TLR9 (*Danio rerio*, NP_001124066), and mouse TLR9 (*Mus musculus*, NP_112455.2).** LRR**,** LRRTYP**,and** LRRCT** domains are indicated with bold letters shaded with yellow accent; transmembrane domains are bold and underlined. CXXC motifs are indicated by black box; highly conserved motifs in TIR domains are double underlined.** - - - -** Sequence gaps,** ∗ **identical residues,**:** conserved substitution,  and. semiconserved substitution.

The full length of RCTLR9B (KF963251) was 3113 bp encoding 1009 amino acid residues. The RCTLR9B protein is homologous to* Rachycentron canadum* isoform A (AGD79973),* Larimichthys crocea* isoform A (ACF60624),* Epinephelus coioides* isoform A (ACV04893), and* Dentex tumifrons* (ABY79218) at 89%, 66%, 65%, and 64%, respectively. A signal peptide located at the 1–29 position of amino acid sequence was identified. In addition, 12 leucine repeats (LRR), LRRTYP (typical), LRRCT (LRR C-terminal), and a 244 amino acid Toll-interleukin-1 receptor (TIR) domain in RCTLR9B were observed. The CXXC motif involved in ligand binding was identified on the LRR motif region at 209–223 amino acid segment containing two conserved motifs separated by six amino acid residues (Figure 1, indicted by a box). Three subsections of TIR domain known to be important in signaling and receptor localization were conserved in RCTLR9A, zebrafish, and mouse TLR9 (Figure 1).

### 3.2. Immune Genes Expression in Juvenile and Adult Intestine

**Figure 2:**
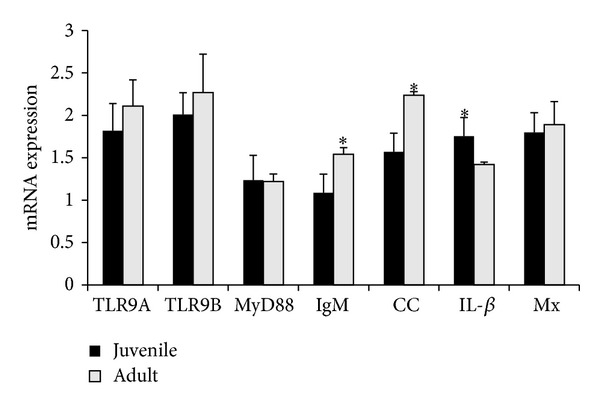
Quantitative analyses of toll-like receptor 9 and immune-related gene expression in intestine of juvenile and adult. The relative expression variance is showed as ratio (the amount of gene mRNA expression normalized to the corresponding beta actin values). Data are shown as mean ± SD (*n* = 2). The significant difference is indicted with asterisk (∗) in the figure.

The mRNA expressions of TLR9 isoform A and B, MyD88, IL-1*β*, IgM, chemokine, and Mx were examined in juvenile and adult cobia (Figure 2). The expressions of IgM and chemokine genes in the adult are significantly (*P* < 0.05) higher than those in the juvenile. The level of IL-1*β* was higher in the juvenile when compared to that in the adult. The expressions of TLR9 A and B isoforms, MyD88, and Mx were not significantly different between the juvenile and the adult.

### 3.3. The Expression of Immune Genes in the Intestine after the Stimulation of CpG ODNs 

#### 3.3.1. TLR 9 A

**Figure 3:**
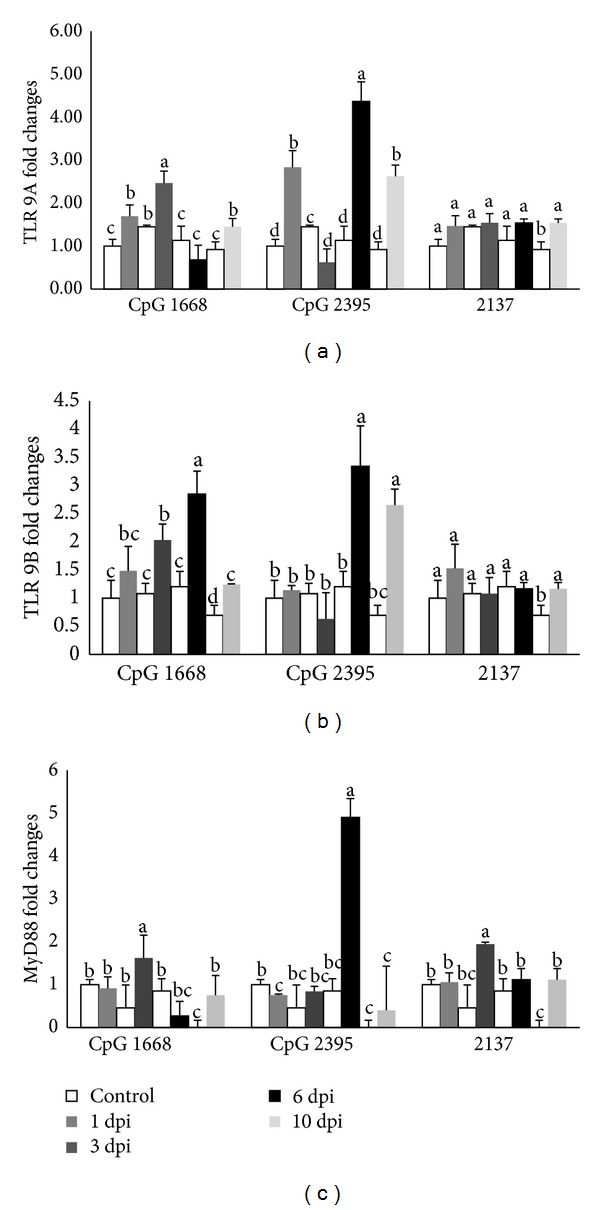
The relative mRNA expressions of TLR 9A (a); TLR 9B (b); and MyD88 (c) in cobia intestine after stimulating with different CpG ODNs (1668, 2395) and ODN 2137 as control measured by quantitative real-tie PCR at 1, 3, 6, and 10 days after stimulation. The expression values represented as “fold change” were compared to the noninjected control samples and *β*-actin was used as a reference gene. The results are presented as the mean ± SD (*n* = 2) and mean values with different alphabetical letters are significantly different (*P* ≤ 0.05).

After CpG ODN 1668 injection, RCTLR9A transcript increased significantly (*P* < 0.05) after 3 dpi, decreased to the lowest level at 6 dpi, and subsequently increased at 10 dpi (Figure 3(a)). While after CpG ODN 2395 injection, the expression bottomed after 3 dpi and sharply peaked at 6 dpi. Among different ODNs, CpG ODN 2395 resulted in the highest fold change of RCTLR9A mRNA in comparison with those of CpG ODN 1668 and CpG ODN 2137.

#### 3.3.2. TLR 9 B

No significant difference of expression was shown in CpG ODN 1668 ODN treated group at 1 dpi (Figure 3(b)). The expression peaked at 6 dpi and later reduced to the lowest. However, the level of RCTLR9B with CpG ODN 2395 stimulation was found to be significantly higher at 6 dpi.

#### 3.3.3. MyD88 Expression

After injection with CpG ODN 1668, the level of MyD88 transcript increased after 1 dpi and peaked at 3 dpi and then reduced to the lowest at 6 dpi and slightly increased at 10 dpi. Significant differences were found at 3 and 6 dpi (Figure 3(c)). The level of MyD88 transcript after injection with CpG ODN 2395 continually increased after 3 dpi, peaked at 6 dpi, and then sharply bottomed at 10 dpi.

#### 3.3.4. Mx Expression

**Figure 4:**
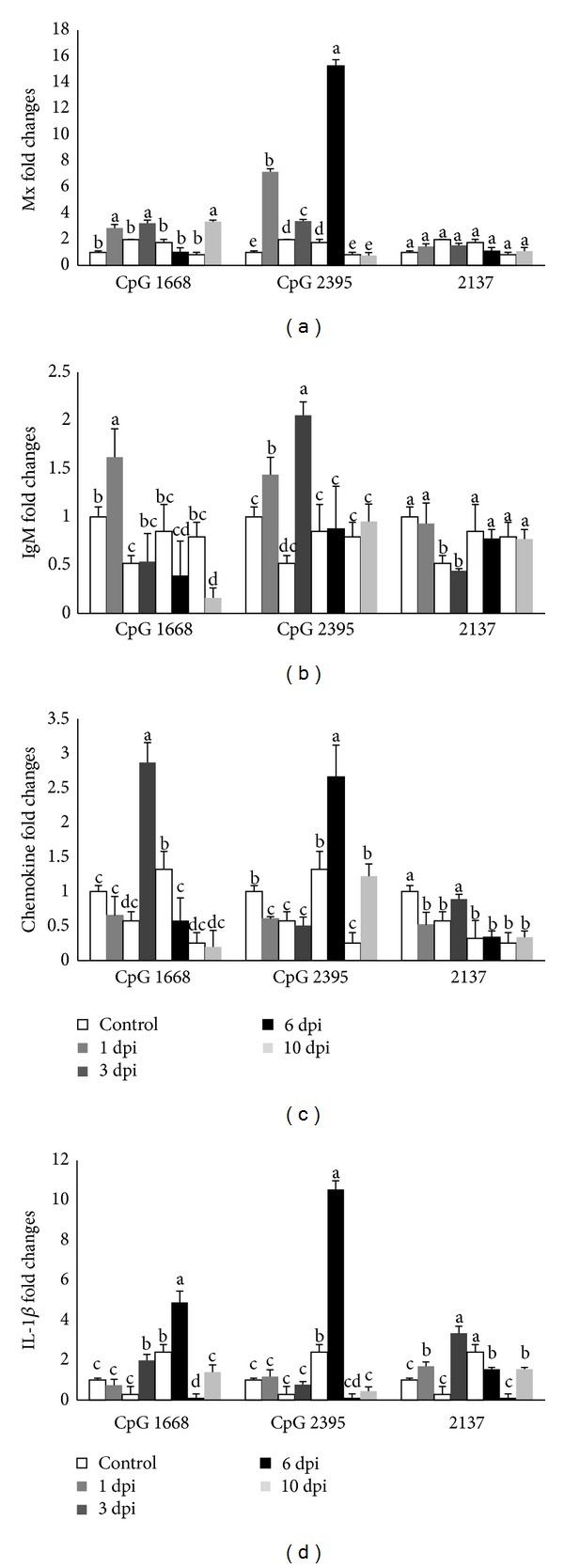
The relative mRNA expressions of Mx (a); IgM (b); CC (c); and interleukin 1-*β* (d) in cobia intestine after stimulating with different CpG ODNs (1668, 2395) and ODN 2137 as control measured by quantitative real-tie PCR at 1, 3, 6, and 10 days after stimulation. The expression values are represented as “fold change” and compared to the noninjected control samples and *β*-actin was used as a reference gene. The results are presented as the mean ± SD (*n* = 2) and mean values with different letters are significantly different (*P* ≤ 0.05).

CpG ODNs 1668 being a class B did not induce high fold change of Mx expression in intestine (Figure 4(a)). However, stimulation with CpG ODNs 2395 has induced significant high expression at 1 dpi and at 3 dpi the expression decreased and later it increased significantly higher at 6 dpi and reduced to the lowest at 10 dpi.

#### 3.3.5. IgM Expression

After CpG ODN 1668 stimulation, IgM transcription peaked at 1 dpi and then decreased at 3 dpi and reached the lowest level at 10 dpi (Figure 4(b)), while the level of IgM transcript with CpG ODN 2395 stimulation increased after 1 dpi and reached the highest level at 3 dpi and sharply reduced at 6 dpi.

#### 3.3.6. Chemokine CC Expression

CpG ODN 1668 has resulted significantly in the increase of chemokine CC and peaked at 3 dpi and then the expression decreased at 6 dpi after injection (Figure 4(c)). The level of chemokine transcript after injection with CpG ODN 2395 bottomed at 3 dpi and reached the highest level at 6 dpi and then dropped at 10 dpi.

#### 3.3.7. IL-1*β* Expression

Expression profile of IL-1*β* in intestine with CpG ODNs 1668 resulted in constant increase after 3 dpi and peaked at 6 dpi; later the expression decreased at 10 dpi (Figure 4(d)). However for CpG ODN 2395 stimulation showed the expression bottomed after 3 dpi and peaked significantly higher at 6 dpi and decreased to the lowest at 10 dpi.

### 3.4. Protection Efficiency of CpG ODNs in Cobia against Bacterial Challenge

**Figure 5:**
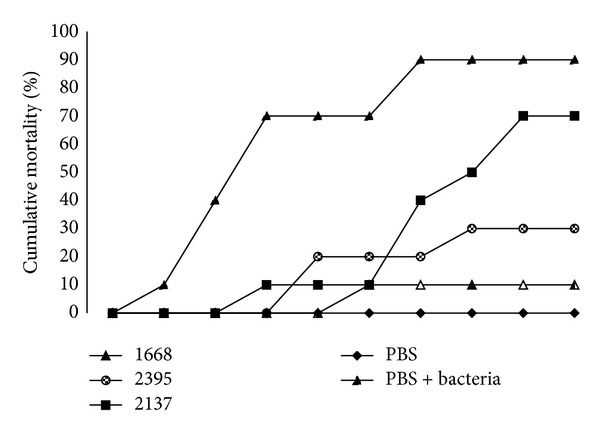
Cumulative percent mortalities of fish injected intraperitoneally (i.p) with 100 µL PBS alone, PBS alone with bacterial challenge, CpG ODN 1668, 2395, and control ODN 2137. After 48 h after CpG ODN injection, cobia was i.p. injected with 50 µL of 3.25 × 10^6^ bacteria (*Photobacterium damselae* subsp.* piscicida*). The group injected with ODN 2395 and 1668 displayed lower mortalities than any other group, *n* = 10 fish per group.

Experiment was conducted to determine whether CpG ODN injected cobia can be protected against bacterial challenge. Results revealed that CpG ODN 1668 and 2395 can protect cobia when challenged with* Photobacterium damselae* subsp.* piscicida* (Figure 5). Fish injected with PBs alone and injected with CpG ODN 2137 began to die at 3 days and 5 days after challenge. Fish injected with PBS showed 90% mortality within 10 days. Among the CpG ODN injected groups, the highest mortality was observed in CpG ODN 2137. The highest survival rate was obtained from CpG ODN 1668 (90%) and followed by CpG ODN 2395 (70%).

### 3.5. The Adjuvanticity of CpG ODNs

#### 3.5.1. TLR 9 Isoform A and B Expressions

**Figure 6:**
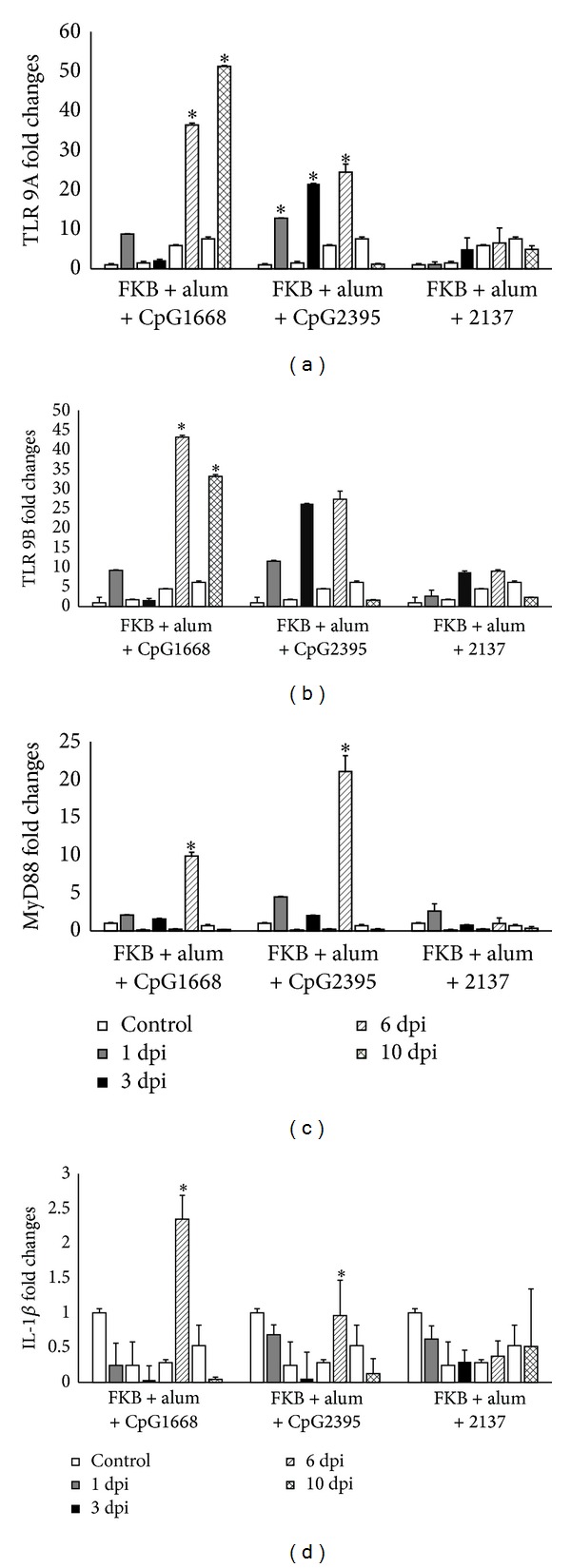
The relative mRNA expressions of (a) TLR9 A, (b) TLR9 B, (c) MyD88, and (d) IL-1*β* in the posterior cobia intestine stimulated with formalin-killed vaccine mixed with CpG and alum and PBS-injected and measured by quantitative real-time PCR. The sampling was carried out at 1, 3, 6, and 10 days after stimulation and *β*-actin was used as a reference gene. The results are presented as the mean ± SD (*n* = 2) and mean values with asterisk (∗) are significantly different (*P* ≤ 0.05).

Both RCTLR9 A and B expressions increased at 1 dpi (~9 and 8 folds) and decreased at 3 dpi after i.p injection of FKB + alum + CpG 1668 (Figures 6(a) and 6(b)). Thereafter, both isoforms A and B increased at 6 dpi (~38 and 42 folds). Expression of isoform A increased at 10 dpi (~51 folds), in contrast, isoform B decreased (~33 folds) during the same time. Expressions of RCTLR 9A and B after being administrated with FKB + alum + CpG 2395 increased significantly until at 6 dpi (~27 and 26 folds) and then decreased at 10 dpi. In control treatment with FKB + alum + 2137, expression of RCTLR9B increased significantly (~5 folds) until at 6 dpi and then decreased at 10 dpi.

#### 3.5.2. MyD88 Expression

Figure 6(c) showed that after administration the expression of Myd88 was fluctuated both in FKB + alum + CpG 1668 and in FKB + alum + CpG 2395. The expression of MyD88 decreased from 1 dpi to 3 dpi and increased significantly at 6 dpi in both CpG-ODN 1668 and CpG-ODN 2395 (~10 and 22 folds) but then decreased and reached the lowest level (~1 fold) at 10 dpi. In control group FKB + alum + 2137, profile of MyD88 showed fluctuated expression. The expression decreased at 3 dpi, increased at 6 dpi, and bottomed at 10 dpi. FKB + alum + CpG 2395 group showed the highest fold changes compared with the other treatment.

#### 3.5.3. IL-1*β* Expression

IL-1*β* expressed low in all three treatments (FKB + alum + CpG 1668; FKB + alum + CpG 2395; and FKB + alum + 2137 treatments) in terms of fold changes. The expression profile of IL-1*β* in both FKB + alum + CpG 1668 and FKB + alum + CpG 2395 became significantly low after 3 dpi. The expression profile of the control group was higher than that of the treatment group in most of observation time (Figure 6(d)). Only at 6 dpi, treatment group showed an increase in expression. In control 2137 group, the expression profile of IL- 1*β* showed fluctuation of no significant difference.

### 3.6. Histological Observation

**Figure 7:**
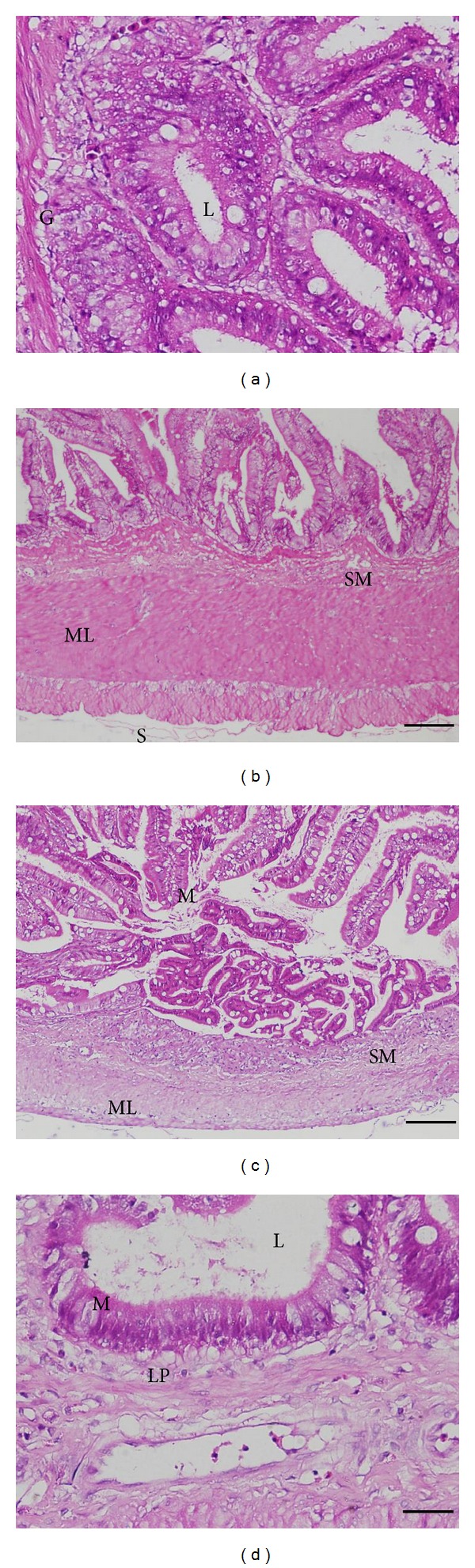
Histopathological observations in posterior part of cobia intestine injected with control PBS (i) and stimulation of cobia mixed with FKB, alum and 2137 (ii), 2395 (iii), and 1668 (iv) at 6 days after stimulation. Letters in the figure are denoted as M: mucosa epithelium, L: lumen, ML: muscular layer, SM: submucosa, LP: lamina propria, S: serosa, and G: goblet cells.

**Figure 8:**
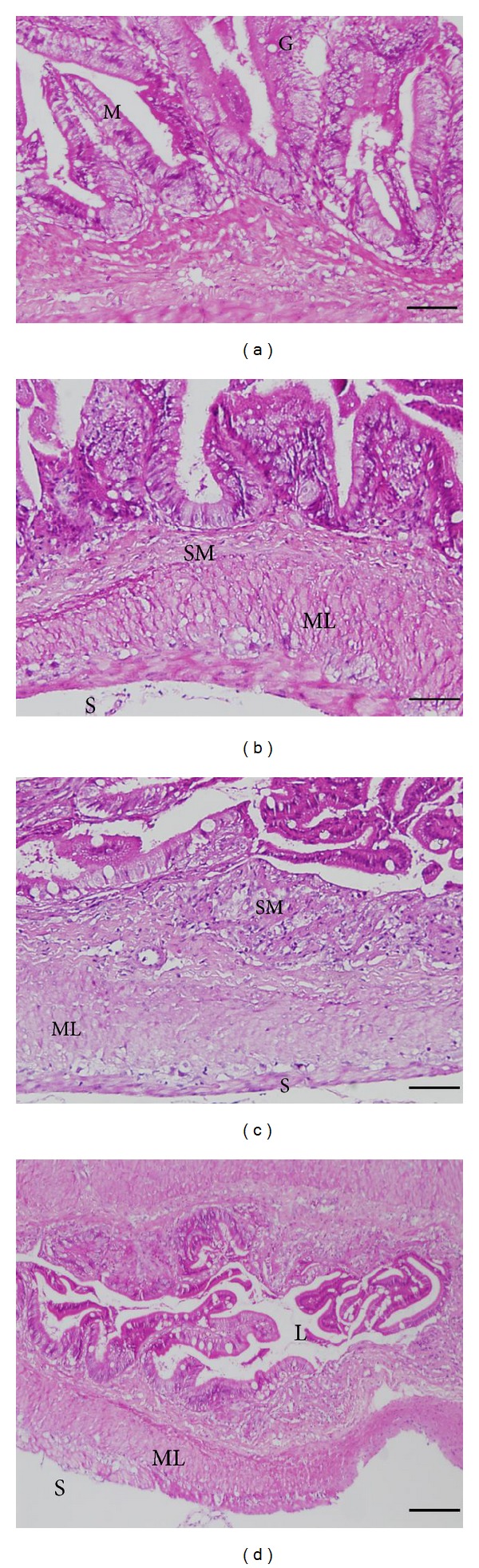
Histopathological observations in posterior part of cobia intestine injected with control PBS (i) and stimulation of cobia mixed with FKB, alum and 2137 (ii), 2395 (iii), and 1668 (iv) at 10 days after stimulation. Letters in the figure are denoted as M: mucosa epithelium, L: lumen, ML: muscular layer, SM: submucosa, LP: lamina propria, S: serosa, and G: goblet cells.

Normal gross morphology on the intestinal wall of cobia was examined using light microscopy (LM) after hematoxylin and eosin (H and E) staining in all groups. No inflammatory cells were found in the lamina propria of the cobia intestine. Additionally, fish from the control group showed an intestinal epithelium consisting of a single layer of columnar epithelial cells with abundant goblet cells joined by apparently intact junctional complexes (Figures 7 and 8). No cell debris was observed in the gut lumen. A continuous mucus layer was evident over the apical surface of the cells. No signs of damage, edema, or inflammation were observed.

## 4. Discussion

RCTLR9B has a transmembrane domain, indicating its localization at the endosomal membrane. The CLUSTALW alignment of cobia (RCTLR9B and RCTLR9A), zebrafish, and mouse TLR9 showed high homology according to the amino acid sequence (Figure 1). Motifs in the cDNA sequence have been proposed to bind to the PAMPs of TLR9 [37]. Our results demonstrated that RCTLR9B has CXXC motifs between 209 and 223 amino acids, suggesting its binding nature to the ligand. In human, five isoforms of TLR9 have been reported (TLR9A–E) [14]. Differential localizations of these TLR9 isoforms in various cells types raise the question of functional significance of computational modeling, structure, and biological relevance a specific isoform may play during inflammation. TLR9 A, C, D, and E are confirmed to be predicted to be located on the ER, but TLR9 B, on the other hand, is located on mitochondria [14]. In teleost, yet only two TLR9 isoforms have been identified in sea bream [10] and croaker [17]. However, the cellular localization of these isoforms in teleost has not been studied.

Both RCTLR9A and RCTLR9B were expressed at a similar level in healthy juvenile and adult cobia. No significant differences in expressions were found between RCTLR9A and RCTLRB or between the juvenile and the adult. This is in agreement with a previous study, in which both TRL9 isoforms were found in the intestine of healthy* Sparus aurata* [10]. However, in juvenile croaker the expression of TLR9B was significantly higher than that of TLR9A [17]. In addition, the expressions of MyD88 and Mx were not significantly different between the juvenile and the adult. Similar levels of expressions were observed in the intestine of healthy rohu [38]. The mRNA expressions of IgM and chemokine CC were significantly higher in adult cobia when compared to juvenile cobia. The basal expressions of CXC and chemokine CC were detected in pig jejunum, caecum, and colon before the treatment of CpG ODNs [24]. The higher expressions of IgM and chemokine CC in adults may indicate the more activated immunity due to the increased exposure to pathogens.

Since the GI tract is the major portal of entry for pathogens [32, 33], it would be meaningful to investigate the expression of genes related to immunity in response to the administration of CpG ODNs in the cobia intestine. The cobia intestinal epithelial cell line is not available; we therefore intraperitoneally injected various CpG ODNs to the fish and acquired intestinal tissues at different time points. Results demonstrated that intraperitoneal stimulation of CpG ODNs had an influence on the expression of genes related to innate immunity in the cobia intestine. The expression of immunity-related genes in the intestine was found to be dependent on the type of CpG ODNs and the time, namely, day after injection (dpi). Fish injected with CpG ODN 2395 had significantly increased expressions of both RCTLR9A and RCTLR9B at 6 dpi, which was reflected on the significantly upregulated expression of MyD88 in comparison with that of fish injected with CpG ODN 1668. A human epithelial cell line, HT-29, was found to spontaneously express TLR9 mRNA and protein, and stimulation with CpG ODNs and bacterial DNA induced the expression of proinflammatory cytokine, including IL-1*β* and IL-8 [39, 40]. In the present study, the expression of IL-1*β* remained unchanged at 1 and 3 dpi, peaked at 6 dpi, and returned to the baseline at 10 dpi. The delayed IL-1*β* response may be due to the following reasons: (1) the activation of TLR9 by CpG ODNs does not always lead to immediate upregulation of an inflammatory cytokine and (2) the presence of TLR9 receptor-ligand interactions leads to intestinal homeostasis which results in the delayed response of inflammatory cytokines [41]. Further investigations would be required to elucidate the relationship between TLR9 and elicited inflammatory responses after the stimulation of CpG ODNs in the cobia intestine.

In response to the stimulation of CpG ODNs, the activated T helper cells draft immunocompetent cells into the intestinal mucosa and influence the chemokines secreted by immune cells, such as macrophages, activated NK cells, and T cells, which determine the subsequent Th1/Th2 immune responses [42, 43]. Intraperitoneal injection of CpG ODN 1668 and 2395 significantly increased the mRNA expression of chemokine CC in the cobia intestine at 3 dpi and 6 dpi, respectively, which might be due to the engagement of immune cells with Th1 type property to the intestinal mucosa after the injection of CpG ODNs. In the piglets administered with CpG ODN D19, the percentage of macrophages and dendritic cells, as well as the expression of chemokine CC, in the intestinal tissue was significantly elevated [24].

CpG ODN 1668 is recognized as a potential immunostimulant for Atlantic salmon, common carp, and Japanese flounder [44–46]. In this study, a CpG ODN other than CpG ODN 1668 and CpG ODN 2395 (a class C CpG ODN) also showed protective effects and significantly increased the survival rate of cobia challenged with live* P. damsela*e subsp.* piscicida*. Similar results found that olive flounder injected with CpG ODN 1668 and 2395 had the highest survival rate against* M. avidus* and* E. tarda* infection, respectively [47, 48]. In the mouse model, administration of CpG ODNs alone has been demonstrated to increase the resistance to listeriosis [49] and* Helicobacter pylori* infection [50]. Oral administration of CpG ODNs protected (90%) newborn mice from* Cryptosporidium parvum* enteric infection [23, 51]. It is difficult to accurately analyze the linkage between the cytokine level and survival rate in aquatic species because only very limited information is available in the literatures. In the present study, the expressions of chemokine CC and IL-1*β* were significantly increased in cobia injected with CpG ODNs. Whether the significantly increased survival rate in cobia injected with CpG ODN 1668 and 2395 was due to upregulated expression of cytokines needs to be confirmed in the future study. However, it has been reported that excessive activation of the immune system with overwhelming production of proinflammatory cytokines can be harmful leading to microcirculatory dysfunction, tissue damage, shock, or even death in severe cases [52]. Moreover, different types of CpG ODNs can elicit different immune responses, for example, the profile of upregulated genes. CpG ODN 2395, being a C-class CpG ODNs, is intermediate between A-class and B-class CpG ODNs in terms of the immunostimulatory activity [53]. The presence of CpG motifs at the 5′-end of C-Class ODN is necessary for inducing strong interferon (IFN)-*α* production [54]. In this region of the CpG ODNs, the required sequence appeared to be similar to that of the B-class CpG ODNs, where the presence of a 5′-TCG is very critical to the immunostimulatory effects [55].

The effect of CpG ODNs on the adaptive immunity of cobia was also explored in the present study. Formulation of CpG ODNs as the adjuvant to enhance the immunogenicity of vaccines has been extensively studied in mice, rabbits, and cattle [56–59]. The combination of FKB, alum, and CpG ODNs (either 1668 or 2395) significantly enhanced the expressions of RCTLR9A and RCTLR9B when compared to the control ODN 2137. Presumably, CpG ODNs initiated the signal transduction in endosomes where TLR9 is located [60–62]. It is noteworthy that the increased expression of TLR9 (~50 folds) in cobia injected with CpG ODN 1688 formulated with alum and FKB was dramatically higher than that of cobia injected with CpG ODN 1688 (~3 folds) alone. This indicated that alum and bacterial antigen (FKB) stimulated the immune responses to upregulate the expression of TLR9, which may facilitate the uptake of the CpG ODNs. CpG ODNs and alum worked synergistically to enhance immune-potentiating and antigen-sparing effects of a vaccine against swine influenza virus [63], which was not observed when CpG ODNs or alum was used alone. The increased expressions (~5 folds) of RCTLR9A and RCTLR9B in cobia injected with the control ODN 213 formulated with alum and FKB may be attributed to the immunostimulatory effects of alum and FKB. In addition, ODNs on a phosphorothioate backbone without CpG motifs have been shown to nonspecifically stimulate TLR9-dependent or -independent activation [64, 65]. The increased expression of MyD88 at 6 dpi implied that the Myd88-dependent signaling pathway of TLR9 was activated in response to CpG ODNs, because this was not seen in cobia injected with the control ODN 2137. The expression of IL-1*β*, a proinflammatory cytokine, was significantly upregulated at 6 dpi in cobia injected with the CpG ODNs, but not the control ODN, with alum and FKB. In a vaccination study on Japanese flounders, the transcription of IL-1*β* was significantly increased in both vaccinated and nonvaccinated groups at 1 dpi indicating that the handling and injecting procedure during vaccination may induce inflammatory responses temporally [66]. Although IL-1*β* has been indicated to upregulate during the course of infections in many mammals, the expression of IL-1*β* in the posterior distal intestine remained unchanged in rainbow trout infected with* A. salmonicida* [67]. In addition, the expression of IL-1*β* may be affected by the amount of antigens present in the posterior intestine of Atlantic cod [68].

Fish vaccines formulated with alum salts are generally accepted due to their safety and satisfactory immunostimulatory effects [69]. In olive flounders, the dosage at 500 *μ*g alum alone per fish only induced very mild inflammation without abnormal histopathological changes [70]. When the dosage was increased to 1600 *μ*g per fish, severe inflammation and mortality were observed. In the present study, only 45 *μ*g alum was used in our vaccines. Moreover, addition of 10 *μ*g CpG ODNs to the vaccine formulation did not cause undesired side effects, such as lamina propria dilation and epithelial cell necrosis, at the site of injection as indicated by the histological observation.

## 5. Conclusion

The cobia TLR9B gene was cloned and ligand binding region CXXC motifs were found on the cobia TLR9B protein. Since TLR9 is the cellular receptor for CpG ODNs, the immunostimulatory effects of CpG ODNs in the intestine were investigated by intraperitoneal injection of various CpG ODNs to cobia fish. Results revealed that the expressions of RCTLR9 and proinflammatory chemokine genes were upregulated and were dependent on the type of CpG ODNs used. The CpG ODNs injected cobia also had significantly increased survival rates after challenge with live* P. damsela*e subsp.* Piscicida*. Finally, the adjuvanticity of CpG ODNs was examined by formulating CpG ODNs as the adjuvant in a vaccine for cobia. The expressions of cobia TLR9, MyD88, and IL-1*β* were significantly elevated in cobia injected with CpG ODNs formulated with alum and FKB. No signs of tissue damage and overwhelming inflammatory responses were observed at the site of injection. Application of CpG ODNs may be used to increase the disease resistance and efficacy of vaccines in cobia.

## Figures and Tables

**Table 1a:** (a) Primers used for cloning of TLR9 isoform B

Primer name	Primer sequence (5′-3′)	Application
TLR9F1^a^	ATCTCAGCCACAATCAGATC	Partial cloning
TLR9R2^a^	AGTTTGGGAAACATCTCATC	

TLR9B1 RacF^b^	CTGTGAATACCCTGAGTCTCAACAGGG	RACE
TLR9B RacR^b^	GTGATGCTGTGAATCAGTACCAGCC	
Universal primer mix (UPM)	Long 0.2 *μ*M CTAATACGACTCACTATAGGGCAAGCAGTGGTATCAACGCAGAGT Short 0.4 *μ*M-CTAATACGACTCACTATAGGGC	
Nested universal primer mix (NUP)	AAGCAGTGGTATCAACGCAGAGT	
TLR9B1-NGSP1	GCCTTTATGTCCTGCCCAAAGCACC	
TLR9B-NGSP2	CTTCCTCGTCTGTTCCATCTTGGCTGT	

**Table 1b:** (b) Primers used for gene expression studies

Primer name	Primer sequence (5′-3′)	Product size (bp)	Target gene	Gene bank accession number	Application
B3-5^c^	ACAGACTGTTCCTCCTCCCC	532	3′UTR	HM754627	cDNA quality
B3-2^c^	GAAACCTCCAACAGCGGG				
RAF	AAGGACCTGTACGCCAACA	330	*β*-Actin-3	HM754627	
RB1	TGGCGTCTCGCATCGTTT				

TLR9A-RC F	TCTGTTCCATCTTGGCTGTG	160	TLR9A	KC180322	qRT-PCR
TLR9A-RC R	CTGGTTTCTGGTGTCAAACA				
TLR9B-RC F	GCCTTCCTCGTCTGTTCCAT	178	TLR9B	JX073035.1	
TLR9B-RC R	ACAGCCTGGTTTCTGGTGTC				
MyD88-F1Q	GAGGTGTAAGAGGATGGTGGT	183	MyD88	KF018033.1*	
MyD88-R2Q	GGTGGGGAATGGCTTTGTCAT				
RCIL-1*β*F1	CAGGCAGAACAACCACTGAC	170	IL-1*β*	AY641829	
RCIL-1*β*R2	TTCCAAGTCCAGTCCTTTGG				
RCMxF1	TGGACATAGCAACCACAGAG	157	Mx	AY834185	
RCMxR2	TTCTTCAGGTGGATGACCTC				
RCIgM-F1	AGACAGCCTGCAGGGAAAAG	182	IgM	JX025102	
RCIgM-R2	TGTTCCTTTCCCCCAGTAGT				
CC1eF	ATTACAATAAGAACCCTGTGC	185	CC	JF975593	
CC1eR	TCTTTCCTGGGATGGATTTG				
*β*-Actin RC-F	ACAGACTGTTCCTCCTCCCC	160	*β*-Actin-3	HM754627	
*β*-Actin RC-R	AAAATCCTGAGTCAAGCGCC				

^a^Partial sequence primers.

^b^Touchdown primers for 5′ and 3′ designed from TLR9 isoform B partial sequence.

^c^Primers designed from 3′UTR of cobia *β*-actin-3.

*Partial sequence cloned in this study.
